# Effect of Winter Cover Crops on Soil Nitrogen Availability, Corn Yield, and Nitrate Leaching

**DOI:** 10.1100/tsw.2001.267

**Published:** 2001-10-25

**Authors:** S. Kuo, B. Huang, R. Bembenek

**Affiliations:** Washington State University, Puyallup, WA 98371, USA

**Keywords:** cover crop, corn, nitrate leaching, nitrogen availability

## Abstract

Biculture of nonlegumes and legumes could serve as cover crops for increasing main crop yield, while reducing NO3 leaching. This study, conducted from 1994 to 1999, determined the effect of monocultured cereal rye (Secale cereale L.), annual ryegrass (Lolium multiflorum), and hairy vetch (Vicia villosa), and bicultured rye/vetch and ryegrass/vetch on N availability in soil, corn (Zea mays L.) yield, and NO3-N leaching in a silt loam soil. The field had been in corn and cover crop rotation since 1987. In addition to the cover crop treatments, there were four N fertilizer rates (0, 67, 134, and 201 kg N ha(-1), referred to as N0, N1, N2, and N3, respectively) applied to corn. The experiment was a randomized split-block design with three replications for each treatment. Lysimeters were installed in 1987 at 0.75 m below the soil surface for leachate collection for the N 0, N 2, and N 3 treatments. The result showed that vetch monoculture had the most influence on soil N availability and corn yield, followed by the bicultures. Rye or ryegrass monoculture had either no effect or an adverse effect on corn yield and soil N availability. Leachate NO3-N concentration was highest where vetch cover crop was planted regardless of N rates, which suggests that N mineralization of vetch N continued well into the fall and winter. Leachate NO3-N concentration increased with increasing N fertilizer rates and exceeded the U.S. Environmental Protection Agency's drinking water standard of 10 mg N l(-1) even at recommended N rate for corn in this region (coastal Pacific Northwest). In comparisons of the average NO3-N concentration during the period of high N leaching, monocultured rye and ryegrass or bicultured rye/vetch and ryegrass/vetch very effectively decreased N leaching in 1998 with dry fall weather. The amount of N available for leaching (determined based on the presidedress nitrate test, the amount of N fertilizer applied, and N uptake) correlated well with average NO3-N during the high N leaching period for vetch cover crop treatment and for the control without the cover crops. The correlation, however, failed for other cover crops largely because of variable effectiveness of the cover crops in reducing NO3 leaching during the 5 years of this study. Further research is needed to determine if relay cover crops planted into standing summer crops is a more appropriate approach than fall seeding in this region to gain sufficient growth of the cover crop by fall. Testing with other main crops that have earlier harvest dates than corn is also needed to further validate the effectiveness of the bicultures to increase soil N availability while protecting the water quality.

## INTRODUCTION

Improving soil quality and crop productivity is central to sustainable agriculture. Winter cover cropping has emerged as a promising practice to help attain this goal. The benefits of winter cover cropping include providing ground cover to protect soil from erosion[1], increasing input of organic matter to soil, and removing the residual soil inorganic N to minimize $NO_3$ leaching and contamination of groundwater[1,2]; however, some winter cover crops are more beneficial than others.

Depending on the region, soil, and weather conditions, different plant species with contrasting chemical characteristics have been used as winter cover crops. Some leguminous cover crops used are hairy vetch, red clover (*Trifolium pratense* L.), and subterranean clovers (*T. subterraneum* L.); nonleguminous cover crops are cereal rye (*Secale cereale* L.) and annual ryegrass (*Lolium multiflorum* L.), among others. When planted after fall harvest, nonleguminous cover crops reduced N leaching by 20 to 69 kg N $ha^{-1}$ depending on the region, cropping system, cover crop species, and the amount of supplemental N applied[2,3,4]. Nonleguminous cover crop residues, however, have no effect or even adverse effects on production of a succeeding main crop[5], possibly because of N immobilization[6] or production of allopathic compounds[7]; thus, while serving an important function of decreasing N leaching and increasing soil organic C and N accumulation, it is not beneficial to the production of a succeeding crop.

Even though legume cover crops are capable of increasing soil N availability and growth of succeeding crops[1,8], they are much less effective than nonlegumes in reducing N leaching[2]. Inclusion of legumes in a crop rotation could even enhance N leaching in legume-based rotation systems[9].

A winter cover crop of legume and nonlegume combination could both enhance soil productivity and reduce $NO_3$-N leaching. The benefit of biculture of rye and hairy vetch to soil N availability and succeeding corn yield has recently been validated in eastern and southeastern regions of the U.S.[10,11]. It further increased with late kill dates of the cover crop[10,12]. How effective the biculture is in reducing $NO_3$-N leaching, and how well it can increase soil N availability in soil and productivity of the main summer crop in the coastal Pacific Northwest region with predominately cool summers and mild winters are not known.

The objectives of this study were (1) to determine N accumulation in various types of cover crops, including monoculture of legume or nonlegume and biculture of legume and nonlegume; (2) to determine the effectiveness of presidedress nitrate N test (PSNT) as a good measure of available N in the soil incorporated with different types of cover crop residues, and (3) to determine the effect of N fertilizer rates and cover crops on N leaching during winter high-rainfall period.

## MATERIALS AND METHODS

The study was conducted on a Sultan silt loam (fine-silty, mixed, mesic Aquic Xerofluent) from 1994 to 1999 at Puyallup, WA. The cover crops used included hairy vetch, annual ryegrass, and cereal rye, and their respective seeding rates for monoculture of the cover crops were 36, 28, and 134 kg $ha^{-1}$. For biculture, the seeding rate for each species was 70% of that of monoculture. Cover crop treatment was the main plot ($9.1 \times 36.6$ m). A control without any of the cover crops was included for comparison. There were four N fertilizer rates (0, 67, 134, and 201 kg N $ha^{-1}$) (referred to as $N_0$, $N_1$, $N_2$, and $N_3$, respectively) as subplots in a split-plot design. The subplot was $9.1 \times 4.6$ m. The site has an average annual rainfall of 97 cm, 75% of which occurs from October to March, and an average annual temperature of 10.6°C.

Each year, after the soil had been tilled with a rototiller to a depth of about 10 cm, the cover crops were seeded between the fourth week of September and first week of October with a row spacing of 19 cm. Small amounts of K (56 kg K $ha^{-1}$ as KCl) and P (18 kg P $ha^{-1}$ as triple superphosphate) were applied to the soil before seeding of the cover crops. The aboveground biomass of the cover crops and of the control crop was determined about the last week of April each year by mowing a strip $2 \times 5$ to $2 \times 9$ m, depending on the cover crop, using a flail chopper. A subsample from each plot was taken for the determination of moisture content by oven drying at 65°C for 72 h and for total N analysis by digestion in concentrated $H_2SO_4$ and $H_2O_2$, followed by steam distillation and titration[13].

After the cover crops were sampled, the soil was moldboard plowed to a depth of about 20 cm, followed by rototilling to a depth of about 10 cm. K (223 kg K $ha^{-1}$) and S (11 kg $ha^{-1}$) were broadcast and incorporated into the soil; 0 or 67 kg N $ha^{-1}$ as $NH_4NO_3$ and 44 kg P $ha^{-1}$ as triple superphosphate were applied in a band 3 cm away from the seed and 6 cm below the soil surface at the time of corn seeding. Pioneer 3845 corn was seeded at the rate of 91,000 seeds per hectare. Additional N fertilizer was sidedressed to some plots to bring the total N fertilizer applied to 134 or 201 kg N $ha^{-1}$. Corn was harvested during the third or fourth week of September each year by cutting all of the plants in one 3.2-m corn row in the middle of each plot. The plants were chopped using a forage chopper, and subsamples were taken for the determination of moisture content and tissue N content using the same procedures described above.

Before sidedressing, soil samples were taken from all plots late in June to a depth of 30 cm in 15-cm increments from midway between the corn rows of each plot. The soil samples were air-dried and crushed to pass through a 2-mm sieve. The soil samples were analyzed for $NO_3$-N by extracting the soils with 2 *M* KCl, followed by $NO_3$-N analysis using an autoanalyzer.

Lysimeters were constructed and installed in the summer of 1987 in the plots where N application rates were $N_0$, $N_2$, and $N_3$ with or without the cover crops. Each lysimeter consisted of a trough made of 150-cm long and 15-cm (I.D.) polyvinyl chloride (PVC) pipe cut vertically through the middle. Each end of the trough was sealed with a piece of plexiglass before it was filled with silica sands and placed 75 cm below soil surface in the middle of each plot perpendicular to the corn rows. One end was raised slightly higher to allow the water to flow by gravitation to the other end and via a high-density Tygon tubing flew into a receptacle situated about 15 cm away and 15 cm below the lysimeter. The receptacle was made of a 30-cm (I.D.) × 30-cm PVC pipe with each end sealed with a PVC cap. An opening of 1 cm (I.D.) on the top cap was drilled, and a small PVC tube was inserted to the bottom of the receptacle. The PVC tube was long enough to allow the tube to reach 25 cm below the soil surface, which was about 5 cm below the plow depth. The tube was connected to a Tygon tubing in the fall after corn harvest and disconnected and capped in the spring before the soil was tilled.

To install the lysimeter, a trench 20-cm wide and 80-cm deep was dug. The soil dug out from each trench was separated by depth. The surface soil (0 to 25 cm) was placed in one pile, the next 25 cm in a second pile, and the rest in a third pile. After the installation of the lysimeter was complete, the soils were put back into the trench, the third pile first, followed by the second and then the first piles. This method of lysimeter installation disturbs the soil profile above the lysimeter and could affect drainage volume. Lysimeter with disturbed soil profile is adequate for comparing relative effect of cropping system on N leaching[14].

Sampling of water inside the receptacle began late in October or early in November. After the first water sampling, water was sampled again after every 5 cm of rainfall. At each sampling, the receptacle was pumped empty before the fresh leachate was sampled. Because the water table may sometimes reach the soil surface during the high-rainfall period in January, the total leachate volume per unit area could not be accurately measured. In this regard, the treatment effect on N leaching was determined by comparing $NO_3$-N concentrations in the leachate.

Analysis of variance was done using the general linear method of SAS[15]. Mean separation was performed using Duncan's Multiple Range test. Regression analysis was used to determine the relationships between corn yield and soil N availability, and between leachate $NO_3$-N and available N in the soil.

## RESULTS AND DISCUSSION

### Cover Crop N, Soil N Availability, and Corn Yield

The average biomass production over 5 years for each cover crop was used for comparing yield potential among the cover crops. It decreased in the order of ryegrass/vetch > rye/vetch > vetch > rye = ryegrass (Table 1). A higher biomass for the bicultures than for the monocultures is to be expected, given higher combined seeding rates for the bicultures than for the monocultures. The average cover crop N concentration decreased in the order of monoculture vetch > bicultured rye/vetch or ryegrass/vetch > monocultured rye or annual ryegrass; the reverse was true for the C:N ratio. The inclusion of vetch with rye or ryegrass raised average N concentration by about 9 to 11 g N $kg^{-1}$ over the monocultured rye or ryegrass and lowered C:N ratios to below 20 (Table 1). In terms of total N accumulation, the bicultures accumulated slightly less than or close to that of vetch monoculture, but much more compared to total N accumulation by monocultured rye or ryegrass. In a study of grass and legume cover crops in the eastern region of the U.S., the bicultures were found to produce more biomass but slightly less total N accumulation than monocultured vetch[11]. The biomass and N uptake by hairy vetch, however, was much lower in this coastal Pacific Northwest region than in the eastern and southeastern regions of the U.S. when harvested late in April[10,11]. The difference could be attributed to regional differences in weather conditions (e.g., temperature).

The PSNT for corn determines the amount of soil available N at the sixth leaf stage[16]. It reflects the overall influence of soil, cover crop residue, and environmental conditions on the net N mineralization in soil early in a growing season. The overall effect of the cover crops on PSNT over 5 years is illustrated by the relationship between the average cover crop N and the average PSNT. The effect was significant ($r^2 = 0.97$, $p < 0.001$). Inclusion of the vetch with rye or ryegrass in bicultures improved the amount of available N in the soil. The average PSNT also correlated well with average C:N ratios of the cover crops and residue in the control plot ($r^2 = 0.88$, $p < 0.001$). Mineralization of organic N in plant residue is determined by the quality of the residue; for example, N concentration or C:N ratio[6]. In monoculture, rye or ryegrass residue lowered soil PSNT to levels below the control (Table 1), possibly as a result of N immobilization[6].

The relationship between PSNT and cover crop N by year was also good with a $r^2$ value ranging from 0.69 to 0.87 (Fig. 1). The response slope, which ranged from 0.92 to 5.31 depending on the year (Fig.1), had an average of 2.61. The slopes of the response of PSNT to cover crop N were lowest in 1996 and highest in 1998. Examination of cumulative monthly rainfall (Fig. 2) showed that during April 1996 it was 19.53 cm (274% of the norm [7.11 cm]), of which 9.19 cm fell within 4 days after cover crop residue incorporation. In contrast, cumulative rainfall for the same month in 1998 was only 35% of the norm. High rainfall, particularly within a few days after residue incorporation, could have created saturation of the soil with water, causing some

**TABLE 1**
**Cover Crop Aboveground Biomass, Tissue N Concentration, C:N ratio, N Accumulation, and PSNT in the Soil Averaged over 5 Years**

| Cover Crop | Biomass (Mg $ha^{-1}$) | N Concentration (g $kg^{-1}$) | N Uptake (kg $ha^{-1}$) | C:N Ratio | PSNT (kg N $ha^{-1}$) |
|---|---|---|---|---|---|
| Control | 0.51 (0.02) | 17.8 (4.3) | 9.1 (0.6) | 21 (1) | 35.2 (25.9) |
| Rye (Ry) | 1.52 (0.54) | 14.4 (1.0) | 20.9 (6.7) | 26 (2) | 31.4 (16.2) |
| Ryegrass (Rg) | 1.57 (0.36) | 12.4 (2.1) | 18.8 (3.6) | 31 (5) | 22.9 (16.3) |
| Vetch (V) | 1.82 (0.37) | 32.6 (3.0) | 58.3 (9.5) | 12 (1) | 69.8 (41.2) |
| Ry/V | 2.18 (0.24) | 23.2 (3.0) | 49.7 (7.0) | 16 (2) | 45.7 (10.1) |
| Rg/V | 2.53 (0.56) | 23.2 (5.9) | 56.3 (9.0) | 17 (4) | 53.0 (32.2) |

*Note*: Values within parentheses are standard deviation.

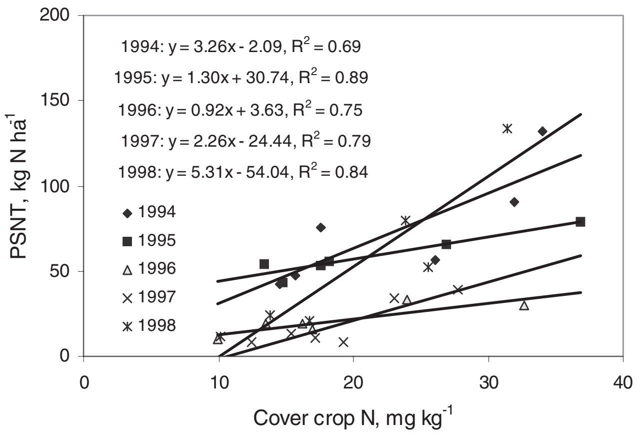

**FIGURE 1.** The relationship between PSNT and cover crop N.

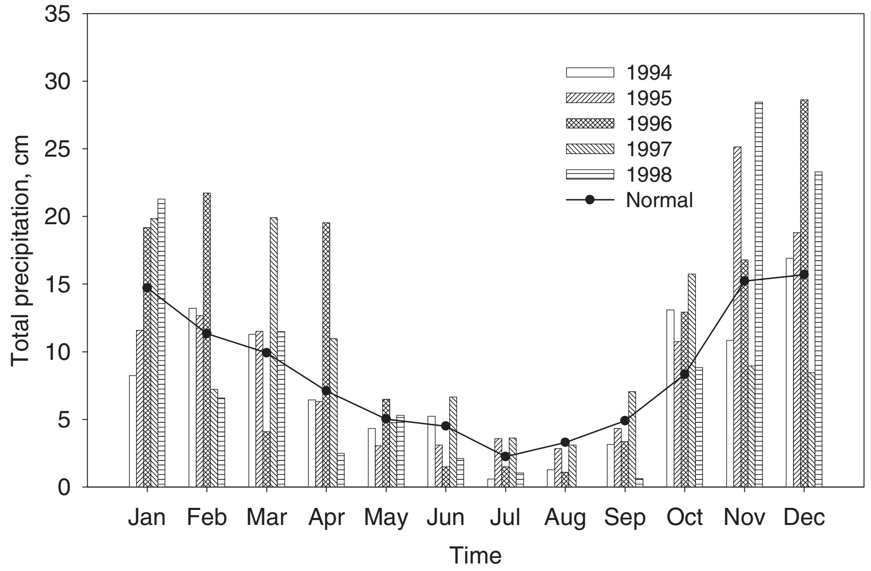

**FIGURE 2.** The mean precipitation by month from 1994 to 1998 in the Puyallup area.

N loss by leaching and/or denitrification. Under the circumstances, PSNT levels in 1997 did not respond to cover crop N in the same manner as in 1998 or the other years. The response slope averaged over 5 years was close to that based on average PSNT and average cover crop N over the same period (2.26). The significance of the relationship between cover crop N on PSNT of the soil was found whether the relationship was evaluated by year or based on the average cover crop N and average PSNT over the 5-year period.

The average corn yield over the 5-year period for cover crop treatments with no N fertilizer addition was highest following vetch (15.2 Mg ha$^{-1}$), intermediate following rye/vetch (13.7 Mg ha$^{-1}$) or ryegrass/vetch (11.1 Mg ha$^{-1}$), and lowest following rye (10.0 Mg ha$^{-1}$) or ryegrass (8.9 Mg ha$^{-1}$). The yield for the control with no N fertilizer addition was 9.4 Mg ha$^{-1}$. For N uptake, the pattern was same as that for corn yield. The average corn N uptake was highest following the vetch (138 kg N ha$^{-1}$), intermediate following rye/vetch (117 kg N ha$^{-1}$) or ryegrass/vetch (91 kg N ha$^{-1}$), and lowest following rye (83 kg N ha$^{-1}$) or ryegrass cover crop (68 kg N ha$^{-1}$). For the control, N uptake was as low as that for monocultured ryegrass (68 kg N ha$^{-1}$). Inclusion of the vetch with rye or ryegrass was beneficial to corn production. Corn yield and N uptake were further increased with increasing N fertilizer rates. The detailed response has been discussed in Kuo et al.[17].

The monocultured vetch or bicultures benefited corn yield and N uptake by increasing soil PSNT. This is demonstrated by the close relationship between PSNT and corn yield (Fig. 3) or N uptake (data not included). The PSNT for corn gave a good index of available N in the soil treated with different cover crop residues. This relationship is important to the assessment of N leaching after the fall harvest.

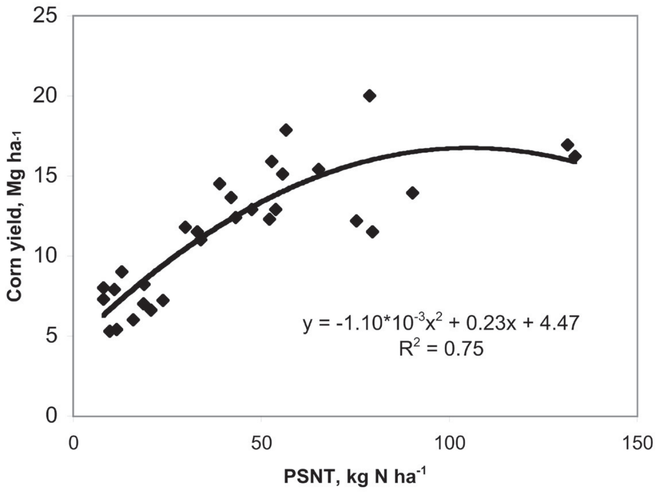

**FIGURE 3**. The relationship between corn yield and PSNT from 1994 to 1998.

## N Leaching as Affected by Cover Crop Species and N Fertilizer Rate

Leachate $NO_3$-N concentration varied considerably with N fertilizer rate, cover crop species, year, and time within year (Fig. 4). Within a year, leachate $NO_3$-N concentration was highest between October and December. It also varied ($p < 0.05$) with N rate. Increasing N fertilizer rate from $N_0$ to $N_2$ increased leachate $NO_3$-N concentrations to above 10 mg N $l^{-1}$ in every year for vetch and in some years for other cover crops during the period of high N leaching. Even at the N fertilizer rate typically recommended for corn production in this region, leachate N can exceed the U.S. Environmental Protection Agency's (EPA's) drinking water standard. Leachate N concentration further increased when N rate was increased to $N_3$. Again, they were highest in the soil where the vetch was used as a cover crop.

A significant year effect on N leaching even in the same soil has been found[2]. Leachate $NO_3$-N concentration during the period of high N leaching averaged across all cover crop treatment at $N_3$, for example, was highest in 1994 (30.5 mg N $l^{-1}$), intermediate in 1995 (21.2 mg N $l^{-1}$) and 1996 (18.0 mg N $l^{-1}$), and lowest in 1997 (7.9 mg N $l^{-1}$) and 1998 (13.1 mg N $l^{-1}$). The highest N leaching in the fall of 1994 (Fig. 4) coincided with a high soil N mineralization early in the growing season (Fig. 1), which increased the pool size of available N in the soil. The lowest leachate N concentration in 1997 coincided with a high precipitation of 15.75 cm during October—close to twice the norm, the average over a period of 30 years (1961 to 1990). Some of residual available N could have already been leached before leachate was first collected late in October. This wet month was followed by substantially lower precipitation in November (8.94 cm) and December (8.46 cm) compared with the norm of 15.21 and 15.70 cm for November and December, respectively. The relatively dry November and December could have promoted the growth of the cover crops, thereby increasing their ability to remove available N from soil.

The concentration of $NO_3$-N in leachate was generally much higher for vetch than for other cover crops or the control at the same N rate. This indicates that mineralization of organic N from vetch continued into winter in this region with mild winters. This brought $NO_3$-N concentration in leachate well over that for other cover crops at $N_2$ or $N_3$ and exceeded the EPA's drinking water standard of 10 mg $NO_3$-N $l^{-1}$. The vetch had little capability to reduce $NO_3$-N and even aggravated it. It is possible that with limited vegetative growth when seeded in the fall, the ability of the vetch to absorb N from soil was limited[18]. Field observations during the period of this study revealed that much of the growth of the vetch did not come until late in March or April, as has been reported in other studies[10,18]. While cover cropping with hairy vetch improved soil N availability and corn growth during the growing season, it also increased N leaching in fall and winter. To increase N use efficiency and to decrease $NO_3$-N leaching, the rate of N fertilizer applied to corn following a vetch cover crop should be adjusted according to the amount of available N contributed from the legume; otherwise, N leaching would be intensified.

Inclusion of rye or ryegrass with vetch in biculture decreased N leaching compared to monocultured vetch. The extent of N leaching for the bicultures was close to that of monocultured rye or ryegrass at the same N fertilizer rate. While the biculture increased soil N availability during the growing season and corn yield, N leaching in the soil was not increased to a level above that of monocultured rye or ryegrass.

The rye or ryegrass in biculture did not consistently reduce $NO_3$-N concentration in leachate to levels below the 10 mg N $l^{-1}$ drinking water standard during the period of high N leaching. During this period, the standard was exceeded one (1994) out of five seasons for rye/vetch and three (1994, 1995, and 1996) out of five for ryegrass/vetch at $N_2$. With a lower frequency of leachate $NO_3$-N exceeding the standard, rye/vetch is preferred to ryegrass/vetch as a biculture cover crop. For rye/vetch the frequency of leachate $NO_3$-N levels exceeding the standard increased from one to three (1994, 1995, and 1996) as N fertilizer rate was fur-

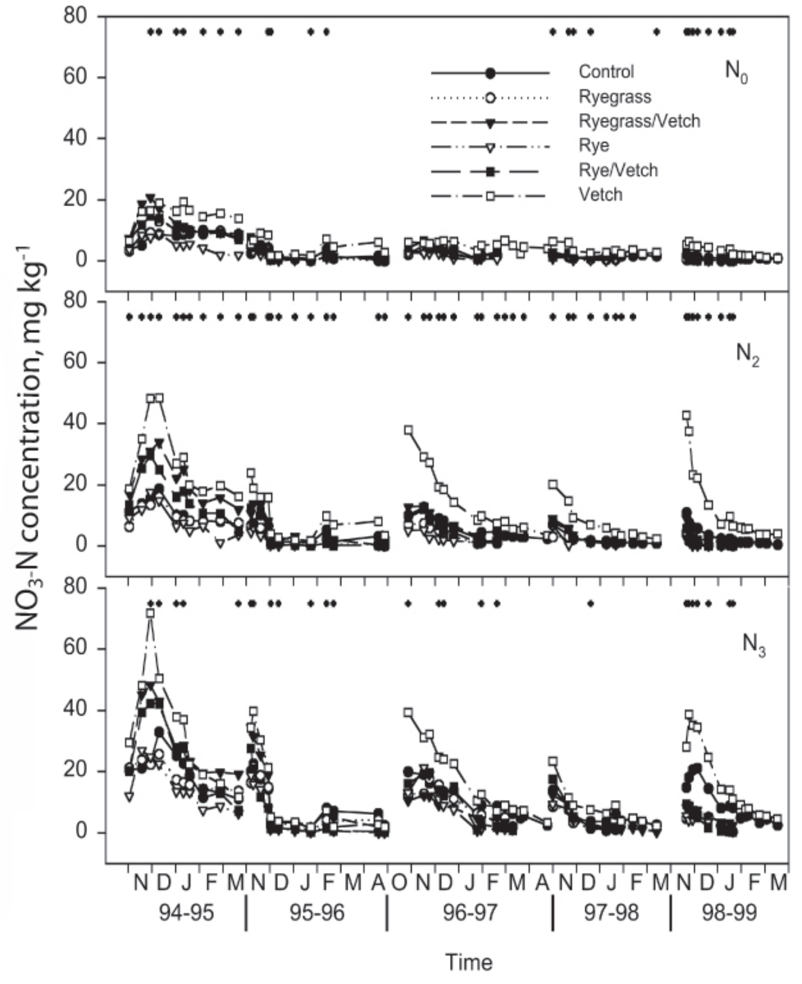

**FIGURE 4.** Leachate $NO_3$-N concentrations as affected by cover crops and N fertilizer rates from 1994 to 1999. Plus symbols at sampling dates indicate significant differences at $p < 0.05$ for the mean comparisons among the cover crop treatments.

ther increased to $N_3$, the same as that for ryegrass/vetch. A limited capability of both bicultures to reduce N leaching in the soil at high N input was seen.

Biculture of rye or ryegrass with vetch and monoculture of rye or ryegrass were most effective in reducing N leaching in 1998. The average $NO_3$-N concentration in leachate in the control during the period of high N leaching in this year was 7.5 mg $l^{-1}$ and 18.7 mg N $l^{-1}$ at $N_2$ and $N_3$, respectively. This result was compared with 1.2 to 4.7 mg N $l^{-1}$ at the $N_2$ and 5.0 to 8.1 mg N $l^{-1}$ at the $N_3$ rate for the bicultures and monocultured rye and ryegrass. This year was marked with the lowest cumulative rainfall (9.45 cm) during the months of September and October compared with the norm (13.23 cm) and cumulative rainfall for the other 4 years (16.23, 15.06, 16.28, and 22.81 cm for 1994, 1995, 1996, and 1997, respectively). It is possible that for the bicultures or monoculture of rye or ryegrass to be effective in reducing N leaching when seeded late in September or early in October in this region, dry and mild fall weather conditions are necessary. These conditions allow residual available N to stay mostly in surface soil and the growth of rye or ryegrass to accelerate, thereby effectively absorbing residual N before the onset of winter rainfall. Cover crop growth in the fall determines its effectiveness in removing available N from the soil[3,19]. Alternatives to fall seeding that increase fall growth of cover crop need to be explored for this region. Relay planted cover crops into standing summer crops has been found to improve cover crop growth in the fall and more effectively reduce N leaching[19]. This technique could be considered for the biculture cover cropping system in further evaluating its effectiveness in reducing N leaching.

Estimating the potential of N leaching in soil is an important step in reducing N contamination of groundwater. Total N mass in the 1.2-m soil profile following fall harvest had been used to estimate N leaching[20]. The method is effective, but not really practical[20].

Unlike the southeastern region of the U.S. where heavy rainfall and N leaching occur during the summer period[2], this region typically has a dry summer. The quantity of N available for leaching in fall is related to N input from N fertilizer applied to the main crop, N mineralization from cover crop residue and indigenous soil organic, and N output (the amount of N removed by the main and cover crops). The PSNT gives an index of the amount of available N early in the growing season, as indicated earlier. The available N input may be assumed to comprise PSNT and the amount of N fertilizer applied. Because it does not include N mineralized during the late growing season, it may be positive or negative relative to corn N uptake. If successful, this simple approach can gauge the soil N leaching potential in the fall early in the growing season when N input via N fertilizer application could be adjusted. Where N mineralization of plant residues added is delayed because of high residue C:N ratios,

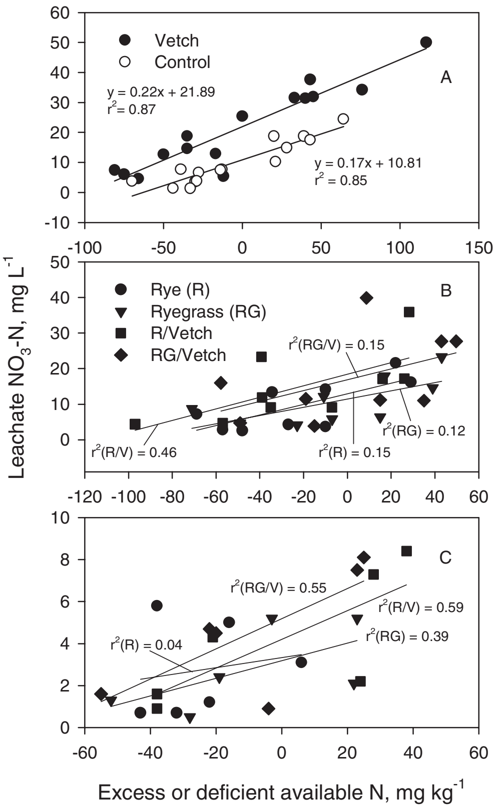

**FIGURE 5.** The relationship between average leachate $NO_3$-N and the amount of available soil N during the period of high N leaching. (A) Vetch and control from 1994 to 1998; (B) monoculture and biculture from 1994 to 1996, and (C) from 1997 to 1998.

however, PSNT may not be a good measure of available N susceptible to leaching and may not be useful for predicting N leaching in fall and winter.

The correlation of average $NO_3$-N during the period of high N leaching in each year with the amount of excess or deficient available N was good for the control and vetch cover crop treatment (Fig. 5A). Vetch cover crop treatment consistently showed a higher average leachate $NO_3$-N over the control during the period of high N leaching throughout the 5 years of this study. The same correlation, however, was poor for either rye/vetch ($r^2$ = 0.28) and ryegrass/vetch ($r^2$ = 0.23) or rye ($r^2$ = 0.33) or ryegrass ($r^2$ = 0.13) (data not included). This low correlation may result from variable effectiveness of these cover crops in reducing N leaching during the 5 years of this study. Segregating 1997 and 1998 with relatively low N leaching from the other 3 years improved the correlation for all but rye cover crop (Fig. 5B and 5C). Given that the low percentage of the variability in N leaching is accounted for by the available N, other factors cause the poor correlation for rye and ryegrass monoculture and rye/vetch and ryegrass/vetch biculture. Further research is needed to determine what these factors are so N leaching can be better estimated using a simple model.

| | | |
|---|---|---|
| Received: | July | 11, 2001 |
| Revised: | September | 24, 2001 |
| Accepted: | October | 3, 2001 |
| Published: | October | 25, 2001 |
